# The current state of prostate cancer treatment in Trinidad and Tobago

**DOI:** 10.3332/ecancer.2018.828

**Published:** 2018-04-24

**Authors:** Satyendra Persaud, Maliza Persaud, Lester Goetz, Dylan Narinesingh

**Affiliations:** 1 Department of Urology, San Fernando General Hospital, San Fernando, Trinidad and Tobago; 2 Division of Clinical Surgical Sciences, The University of the West Indies, St Augustine, Trinidad and Tobago; 3 National Radiotherapy Centre, Port of Spain

**Keywords:** prostate cancer, Trinidad and Tobago, Caribbean

## Abstract

Prostate cancer mortality in the Caribbean region is among the highest in the world and prostate cancer is the most common cancer in Trinidad and Tobago. There is a two-tiered healthcare system in Trinidad and Tobago, and prostate cancer related issues account for a significant percentage of urologists’ workload. Delivery of care is sometimes constrained by limited financial resources. Prostate-specific antigen testing is widely available but there is no national guideline. Treatment options available include active surveillance, radical prostatectomy, external beam radiotherapy and brachytherapy. Patients have access to androgen deprivation, chemotherapy and palliative care for the management of advanced disease. Generally, the infrastructure for treatment in Trinidad and Tobago is satisfactory but would benefit from further investments in technology and human resources.

## Introduction

Trinidad and Tobago, a twin island English speaking republic, are the southernmost islands of the Caribbean, located about 11 km off the north-eastern coast of Venezuela. The most recent decennial Population and Housing Census conducted in 2011 documented a population count of just over 1.3 million spread over the country’s 5,128 km^2^ [1]. Trinidad and Tobago is well known for its ethnically diverse population with the two dominant groups deriving from South Asian and African heritage. Approximately 37.6% of the country’s inhabitants are Indo-Trinidadians while the second largest group, the Afro-Trinidadians and Tobagonians, account for about 36.3%. The remainder of the population consists of persons of mixed descent, European, Chinese and Middle Eastern ancestry. Tobago comprises approximately 300 km^2^ or 6% of the surface area of the country and is unique in that 95% of the population are of African descent [1].

According to data from the Elizabeth Quamina National Cancer Registry of Trinidad and Tobago, prostate cancer accounted for 22% of all cancers in the country, making this the commonest cancer overall as well as the commonest cancer in men. An incidence rate of 60.4/100,000 was documented for the period 2000–2002 and accounted for 39% of all cancer related mortality among men [Data–National Cancer Registry]. Mortality from prostate cancer has been previously described as among the highest in the world [2]

There is established evidence of a racial disparity in prostate cancer in Trinidad and Tobago. Mungrue found that prostate cancer was almost five times as common among Afro-Trinidadians. In this study, Afro-Trinidadians had higher Gleason scores, prostate-specific antigen (PSA) values and ultimately an increased risk of mortality [3]. Recent work at the San Fernando General Hospital (SFGH) reported that prostate cancer was three times as common among Afro-Trinidadians compared to Indo-Trinidadians. Afro-Trinidadians were more likely to have poorly differentiated tumours, high PSA values and D’Amico high-risk cancers [4].

Prostate cancer management requires a multimodal approach and involves the collaborative effort of our urologists, pathologists, medical and radiation oncologists, and palliative care physicians. The following article details the current situation with regard to prostate cancer screening, treatment and outcomes in Trinidad and Tobago.

## Human resources and prostate cancer treatment

There is a two-tiered healthcare system in place in Trinidad and Tobago—public and private. While the government–run Ministry of Health is ultimately responsible for the provision of free health care for the general public, it has devolved into several separate entities that are labelled as regional health authorities (RHAs). Resources are allocated by the Ministry to these RHAs to finance their operations to meet goals and targets set by the Ministry based on an assessment of health needs of each region. The main hospitals on the island of Trinidad are Port of Spain General Hospital (POSGH), SFGH, Eric Williams Medical Sciences Complex (EWMSC), Sangre Grande Hospital and Point Fortin Hospital. Scarborough General Hospital is located in Tobago and serves as the main healthcare facility for Tobagonians. The largest urological unit is located at the SFGH which houses 24-bed Urology Ward as well as two dedicated urology theatres—this hospital is certified as an International Society of Urology approved training facility and is staffed by four consultant urologists. Urological services are also available at the EWMSC and POSGH with both units having two consultant urologists each. There is no consistent urological service at the Sangre Grande or Point Fortin Hospitals.

The skewed distribution of urological services across the country has resulted in substantial difficulties in providing optimal health care with regard to prostate cancer screening and treatment. Various private healthcare facilities that are widely distributed across the geography of the country also provide urological care by urologists that are employed solely in the private sector, as well as those who practice in both the private and public sectors. Inclusive of two urologists who have recently completed training, there are 14 practicing urologists in Trinidad and Tobago—three of whom are solely in private practice

The University of the West Indies commenced a post-graduate residency training programme in urology in 2006. The programme is 5 years long with the first 2 years focusing on core surgical rotations. At the end of residency, and following successful completion of examinations, the candidate is awarded the Doctor of Medicine in Urology. In 2015, the first locally trained urologist graduated from the programme and so far, four urologists have completed training. There are presently ten residents at various stages of training. The primary training centre is the SFGH. However, in 2015 another centre, EWMSC in the north of the island was approved by the University of the West Indies. The ratio of urologists to male patients in the population presently stands at 1:47000. With the continued training of residents, there will hopefully be an increase in the number of urology care providers with a more homogeneous dissemination of services throughout the country and a resultant improvement in the management of the large numbers of prostate cancer patients.

The Trinidad and Tobago Urological Association was created on the 13 February 1998 with Dr. Lester Goetz and Dr Hassan Khan the inaugural President and Vice President, respectively. Subsequent to this, on 17 April 1999 at the Cascadia hotel in Trinidad, the Caribbean Urological Association was formed. Dr Lester Goetz was elected as its first President and Dr Hope Russell (Jamaica) was its first Vice President. The secretariat of the Caribbean Urological Association is based at the SFGH in Trinidad.

In terms of nursing support services for patients, while each unit is staffed by what amounts to dedicated urology nurses, there are currently no trained clinical nurse specialists or advanced nursing practitioners to the best of the authors’ knowledge.

## Screening and prostate cancer in Trinidad and Tobago

Even though the prostate cancer screening remains a topic for hot debate, there is no doubt that PSA screening has resulted in a substantial downward migration of prostate cancer stage over the past two decades in the United States [5]. Screening by a combination of digital rectal examination (DRE) and PSA testing is recommended by many experts since a proportion of clinically significant cancers may be potentially missed by utilising PSA alone [5]. The United States Preventative Services Taskforce issued a recommendation against prostate cancer screening, noting that the harms outweigh the benefits [6]. They have recently softened their stance on screening, recommending an individualised approach among men aged 55–69 based on patient–clinician discussion [7]. However, given the disproportionate representation of blacks in these studies as well as the well described heightened risk of prostate cancer in males of African heritage, there have been concerns over the external validity of these recommendations in a Caribbean population and most Caribbean urologists support PSA screening in the region [8].

No formal screening recommendation exists for Trinidad and Tobago. Screening protocols are similar in Trinidad and Tobago, with men at high risk based on ethnicity and family history, being encouraged to have an annual DRE and serum PSA done by the age of 45–50 years.

PSA testing is widely available in Trinidad and Tobago and biopsy services may be accessed via the three major centres—SFGH, EWMSC and POSGH as well as most major private institutions. Initially, single core digitally guided prostate biopsies were done in the absence of an ultrasound machine. However, 12-core transrectal ultrasound guided biopsies are currently the standard, which has significantly improved the biopsy technique and our detection rates. To the best of our knowledge, magnetic resonance imaging (MRI)-guided biopsies of any kind as well as template biopsies are not presently carried out in Trinidad and Tobago.

A study of various sociodemographic groups of males aged 19–60 years in Trinidad and Tobago revealed that a vast majority were aware of the need for prostate cancer screening, especially at older ages [9]. Despite this, men were unwilling to utilise these services, citing apprehension about the DRE and the negative implications of being diagnosed with prostate cancer [9]. Specific DRE related concerns revolved around its association with homosexuality and an ‘assault on manhood’, leading to the assumption that the major barrier to prostate cancer screening in this country is primarily cultural belief and not necessarily a lack of knowledge [9].

Following a needs assessment in 1995, the Tobago Health Services Unit in collaboration with the University of Pittsburgh established a prostate cancer screening study in Tobago [10, 11]. The prevalence of prostate cancer was 10% among the 2484 men screened [12]. The incidence of prostate cancer in Tobago has been estimated to be higher than that among African Americans or Caucasians [10]. The Tobago Prostate Cancer Screening Study has also investigated several metabolic and genetic aspects of prostate cancer including the role of human herpes virus 8, chemoprevention with lycopene and the role of genetic influences such as the ELAC2 and RANSEL genes [10]. The contribution of the late Dr Alan Patrick to this project cannot be overstated.

## Treatment of organ confined prostate cancer in Trinidad and Tobago

The standard recommended treatment modalities for localised prostate cancer—active surveillance, radical prostatectomy, interstitial prostate radiation/brachytherapy and external beam radiation—are all available and widely practiced in Trinidad and Tobago. At present, high intensity focused ultrasound, radiofrequency ablation and cryotherapy are not available in Trinidad and Tobago.

A review of the uro-oncology database of the SFGH revealed that from 2010 to 2017, 5.1%, 1.9%, 34.7%, 48.2% and 10% of men were treated with radical prostatectomy, brachytherapy, external beam radiotherapy, hormonal therapy and active surveillance, respectively [13]. The high proportion of men being treated primarily with hormonal therapy is not surprising given the relatively high number of Trinidadian men presenting with advanced disease compared to the developed world [4]. This trend is, however, changing slowly as a similar review of patients between 2002 and 2009 showed that 80% were treated with hormonal therapy.

### Active surveillance

Active surveillance for prostate cancer is practised in Trinidad and patients are generally selected based on Epstein's criteria [4]. These patients should be classified as low-risk, low-volume prostate cancer with clinical stage of T1- T2a, PSA ≤ 10 ng/dL, Gleason score ≤ 6, <3 cores positive with ≤50% of core involvement and PSA density ≤ 0.15ng/mL. The aim of active surveillance is to avoid the overtreatment of clinically insignificant cancers and with it the adverse effects of prostate cancer treatment. There is concern about the application of active surveillance to men of African descent and it is therefore still unclear whether active surveillance is a safe option for our Caribbean men [15]. Active surveillance is an option to patients throughout the country although there is no uniformity and protocols are at the judgment of the treating urologist. At the SFGH, active surveillance is offered to our low risk patients and a small study of patients enrolled in a single consultants practice. Twenty-four patients have completed 2 years of follow-up and of whom 12 have had repeated biopsy—pathological upgrading was noted in one patient with three reports still outstanding [16]. These findings have hinted that active surveillance may be a safe option for our patients but further study is needed.

### Radical prostatectomy

Open nerve sparing radical retropubic prostatectomy as described by Walsh and colleagues in 1980s, is offered to eligible surgical candidates [17]. Currently, the procedure is offered at the three major hospitals (SFGH, EWMSC, POSGH) as well as the Scarborough General Hospital in Tobago. In the case of Tobago, radical prostatectomies are performed by a Trinidadian urologist who has a visiting arrangement with Tobago. Radical prostatectomies are carried out at several private institutions. The cost is still provider-dependent as fee structures are not currently regulated.

Data from the SFGH reviewing the outcomes of radical prostatectomy over a 12-year period reported an average patient age of 67 years [18]. Oncological outcomes were comparable with other internationally reported figures, with a 5-year biochemical recurrence-free survival of 70% and mean time to biochemical failure of 41 months. Postoperative complications of new onset erectile dysfunction and persistent incontinence were documented at rates of 70% and 12%, respectively. These data are compared to the work done by Morrison and colleagues in Jamaica which demonstrated a 5-year biochemical free survival of 78.4% [19].

Although laparoscopic radical prostatectomies were demonstrated to urologists at the SFGH via several mentored workshops, the procedure has never caught on likely due to the prolonged learning curve required to achieve competence in the procedure. Robotic prostatectomy is unavailable in Trinidad and Tobago.

### Brachytherapy

Brachytherapy involves the insertion of radioactive seeds into the prostate and is done under X-ray and ultrasound guidance [5]. Treatment of prostate cancer with brachytherapy in Trinidad and Tobago is available to patients with low-risk prostate cancer. Brachytherapy is used as the monotherapy treatment with a permanent seed implant. Iodine 125 (I 125) is the source utilised and the dose prescribed is 145 Gy. Patients in Trinidad and Tobago access brachytherapy either privately or through a public–private partnership although the latter is currently on hold. The first brachytherapy patient was treated in 2001 and 241 patients were treated between 2007 and 2012. In this series, there were two cases of biochemical relapse. Complication rates have been in keeping with those reported in the literature with 24% of patients having grades 1 and 2 urinary dysfunction (personal communication).

### External beam radiation

In Trinidad and Tobago external beam radiation is the most common modality used in the treatment of prostate cancer. Radiotherapy is used both as primary therapy and as salvage following radical prostatectomy. Palliative radiotherapy is also widely utilised and is discussed below.

Patients requiring definitive radiotherapy are treated on one of three linear accelerators (linacs) in the island. Two linacs are at the Southern Medical Clinic (SMC) and one is at the Brian Lara Cancer Treatment Centre (BLCTC) in the south and north of the island, respectively. Patients at SMC are treated either with volumetric modulated arc therapy or intensity modulated radiotherapy technique (IMRT) techniques and patients at BLCTC are treated with IMRT. Of the 94 patients referred to the SMC for treatment between the periods 2010–2012, four patients have biochemical relapse and there were 2.1% cases with grade 2–4 genitourinary and 2.8% cases with grade 2–4 gastrointestinal complications (personal communication). Patients requiring treatment at either of these centres may do so privately or through the Ministry of Health programme where the full cost of treatment is borne by the government. Therefore, 100% of patients from 2009 to present with prostate cancer requiring definitive radiation in Trinidad and Tobago will receive treatment with an IMRT technique (or more complex) with the prostate dose taken to at least 74 Gy. Traditionally, patients with intermediate and high-risk prostate cancer have the regional nodes treated.

Patients with intermediate and high-risk prostate cancers receive by protocol, neoadjuvant hormonal therapy along with concurrent and adjuvant treatment with high risk patients receiving at least a total of 3 years of deprivation. Androgen deprivation is almost always in the form of the luteinizing hormone releasing hormone (LHRH) agonist Goserelin.

The average wait-time for patients from the processing of applications by the Ministry of Health to the first scheduled radiotherapy session is about 6–8 weeks. This is a relatively short waiting period considering that these facilities are utilised by all cancer patients across the country and not only for prostate cancer. In one Canadian study, a median treatment interval of 127 days was reported from the biopsy diagnosis of prostate cancer to the first fraction of radiotherapy, a far cry from the recommended 4-week interval by the Canadian Strategy for Cancer Control [20].

## Treatment of advanced prostate cancer

### Androgen deprivation

Surgical castration in the form of bilateral orchidectomy was the original method of androgen deprivation and is still widely performed today. Given the potential psychological impact of orchidectomy as well as the emerging role of intermittent androgen deprivation therapy many men have opted for chemical castration [21]. The most common agents used for androgen deprivation in Trinidad and Tobago are the LHRH analogue goserelin along with the antiandrogens bicalutamide and cyproterone acetate. The LHRH antagonist degarelix was launched in the island in 2013 but is not currently in widespread use.

Androgen deprivation may be used in two settings either as part of definitive treatment with radiation or in the palliative setting. In the definitive treatment with radiation, androgen deprivation is almost universally achieved by a LHRH agonist, e.g., goserelin as outlined above. In the palliative setting androgen deprivation may be achieved by an LHRH agonist or by an orchidectomy. All major public hospitals in Trinidad and Tobago carry out subcapsular orchidectomy as a day-case procedure under local anaesthesia. Combined androgen blockade is offered on progression. Similarly, goserelin is available in the public sector free of charge.

## Castrate resistant and metastatic disease

Patients with castrate resistance are usually offered docetaxel on biochemical progression. Chemotherapy can be accessed at the San Fernando Hospital, Sangre Grande Hospital, National Radiotherapy Centre as well as the Scarborough Hospital in Tobago. Abiraterone is available but is presently not funded by the ministry of health and therefore patients requiring this drug must do so by their own purchase. A combination of other drugs is tried on progression including ketoconazole and diethylstilbestrol.

Technetium bone scintigraphy is available in the private sector. Public patients requiring scans are funded by the Ministry of Health to have the scans done in these private facilities.

## Palliative care

Palliative care in Trinidad and Tobago can be accessed by all nationals free of charge. There are three hospices in Trinidad and Tobago, two are run by non-governmental organizations and one is run by the state. All major public hospitals have associated palliative outpatient clinics or community palliative services. The Trinidad and Tobago government in 2014 signed on to the United Nations resolution recognising palliative care as a human right. Since then, the Ministry of Health has worked to regulatory and technical support for palliative care. The University of the West Indies in 2013 started a Master’s programme in Palliative Medicine. The graduates from this programme have begun to service the palliative care units in the island. The admission rate for end of life events at the SFGH dropped from 85% to 20% in 1 year following the introduction of a palliative care service.

The Palliative Care Unit in Caura is a 12-bed facility which was opened in 2014 and offers treatment on an in and out-patient basis. Vitas House in St James was opened in 2008 and also has 12 in-patient beds as well as an out-patient palliative care clinic. Vitas House accepts patients with a life expectancy of 6 months or less and is staffed by two doctors and one nurse along with administrative staff and volunteers. Prior to the commissioning of the facilities at Caura and Vitas House, the Living Water Hospice offered care for terminally ill patients at their 16-bed facility which was opened in 1983. Operated by the Catholic community, this facility is heavily dependent on volunteers.

Because of inconsistency in the availability of interventional radiology and in the face of great need for the procedure, urologists at the SFGH acquired the skills to provide nephrostomy tubes for urinary diversion in the case of malignant ureteric obstruction in advanced prostate cancer. Currently, this service is offered on a regular basis at the SFGH and on a very limited basis at the EWMSC where it is primarily radiologist led.

In the case of malignant spinal cord obstruction, neurosurgical services are available at all major hospitals and emergency radiotherapy is efficiently coordinated by the oncologists. Patients requiring palliative therapy are treated either privately with a linear accelerator or in the public service with a cobalt machine. An audit done at the National Radiotherapy Centre (public hospital) revealed the average time to treatment for palliative cases was 3 hours in 2015.

## Conclusion

Prostate cancer remains a significant public health concern in Trinidad and Tobago. While patients have access to PSA-based screening, there is no national policy or coordination on screening. More recent developments in diagnosis such as the use of MRI or newer biomarkers have yet to be adopted on any significant scale. Urological training at the University of the West Indies should improve patient to provider ratio and lead to more uniform access to care. While all men have access to treatment with curative intent, there remains significant room for improvement.
